# Beyond Volumization: Repositioning Hyaluronic Acid Fillers as Anatomy‐Guided Structural Support Tools in the Era of Biostimulators

**DOI:** 10.1111/jocd.71179

**Published:** 2026-09-01

**Authors:** Tara Margarella, Seung Jun Shin

**Affiliations:** ^1^ Department of Medical Affairs Hugel Aesthetics Irvine California USA; ^2^ Department of Medical Affairs Hugel, Inc Seoul Republic of Korea

**Keywords:** facial aging, facial anatomy, hyaluronic acid filler, retaining ligaments, rheology, structural support, vascular safety

## Abstract

**Background:**

Hyaluronic acid (HA) fillers remain widely used in facial rejuvenation, yet concern about overfilled syndrome and unnatural volumization has shifted attention toward biostimulators. These concerns may reflect a mismatch between volumetric treatment logic and structural problems of facial aging.

**Objective:**

To propose an anatomy‐guided framework that repositions HA fillers from default volume replacement toward selective structural support and lifting‐oriented treatment planning.

**Methods:**

A targeted narrative review of anatomical, clinical, rheological, biological, and vascular‐safety literature was conducted using PubMed, Google Scholar, and relevant journal databases through July 2026.

**Results:**

Facial aging involves skeletal remodeling, fat‐compartment changes, dermal deterioration, and altered relationships among retaining ligaments and mobile soft tissues. Ligament‐associated HA injection is best interpreted as mechanical scaffold augmentation in selected periosteal or periligamentous support zones rather than injection into the ligament itself or ligament regeneration. Current evidence supports the plausibility of support‐mediated contour improvement but does not establish true tissue repositioning independent of projection or volumizing effects. Product selection should consider plane, function, G′, cohesivity, viscosity, swelling tendency, and vascular risk rather than G′ alone. HA and biostimulators have complementary and overlapping roles.

**Conclusion:**

HA fillers should not be viewed as obsolete because of overfilling. Appropriate patient selection, anatomy‐guided planning, conservative dosing, region‐specific product selection, and vascular safety may allow HA to function as a structural support tool. The future of HA filler treatment is not less HA, but better‐directed HA.

## 1 Introduction

Hyaluronic acid (HA) fillers occupy a paradoxical position in contemporary aesthetic medicine. They remain among the most widely used injectable materials because of their precision, reversibility, broad range of rheological profiles, and extensive clinical experience. At the same time, skepticism toward HA fillers has increased in parallel with the rise of biostimulators, regenerative concepts, and treatment philosophies emphasizing naturalness, restraint, and tissue quality. This skepticism is not without basis. Excessive, poorly sequenced, or anatomically insensitive HA filler use may contribute to overfilled syndrome, distorted surface contours, facial heaviness, and cumulative aesthetic disharmony [1].

However, the limitations attributed to HA fillers may often reflect a mismatch between the treatment problem and the conceptual framework used to address it. HA fillers became dominant during an era in which facial aging was frequently interpreted through the lens of volume loss. Within that framework, volume replacement became the default response to aging‐related depressions, folds, and contour changes. Yet facial aging is not purely volumetric. It involves progressive changes across multiple anatomical layers, including skeletal remodeling, fat compartment deflation and redistribution, dermal thinning, altered tissue elasticity, and changes in ligament‐associated support systems [2, 3, 4, 5, 6]. When HA fillers are applied to structural problems using only volumetric logic, the result may be increased fullness without restoration of youthful facial geometry.

The central argument of this review is that contemporary skepticism toward HA fillers should not be interpreted simply as evidence that HA fillers have become obsolete in the era of biostimulators. Rather, it reflects the limitations of using HA fillers primarily as volumizing agents for problems that are often structural, positional, and support‐related. Overfilled syndrome, facial heaviness, and unnatural broadening are not inevitable consequences of HA as a material; they are frequently consequences of inappropriate indication, excessive medial filling, poor sequencing, and insufficient analysis of the ligament‐fat compartment support system. Therefore, the future role of HA fillers should be repositioned from default volume replacement toward selective, anatomy‐guided structural support. In this framework, HA fillers are not used to fill the aging face indiscriminately, but to support defined anatomical zones where mechanical reinforcement may improve facial contour, reduce reliance on central volumization, and contribute to lifting‐oriented treatment planning in selected patients. Throughout this review, the terms support‐oriented and lifting‐oriented are used interchangeably to describe this anatomy‐guided treatment philosophy.

The purpose of this review is not to provide a step‐by‐step injection manual, fixed injection points, or standardized dosage recommendations. Rather, it is to propose a conceptual and clinically applicable framework for repositioning HA fillers in contemporary facial rejuvenation. Specifically, this review reevaluates the role of HA fillers in response to the limitations associated with indiscriminate volumization and overfilled syndrome, and proposes a treatment framework in which structural support and lifting‐oriented planning are guided by retaining ligament anatomy, fat compartment mechanics, filler rheology, and vascular safety. Exact injection points, volumes, devices, and technical approaches must be individualized according to patient anatomy, tissue quality, prior procedures, product characteristics, injector experience, and vascular risk.

## 2 Methods: Literature Search Strategy

A targeted narrative literature review was conducted to identify anatomical, clinical, rheological, biological, and safety‐related evidence relevant to HA filler use in relation to facial retaining ligament anatomy. Searches were performed using PubMed, Google Scholar, and relevant journal databases through July 2026. Search terms included combinations of “hyaluronic acid filler,” “dermal filler,” “facial retaining ligaments,” “line of ligaments,” “zygomatic ligament,” “mandibular ligament,” “temporal ligamentous adhesion,” “lateral orbital thickening,” “facial fat compartments,” “facial aging,” “G prime,” “cohesivity,” “filler rheology,” “vascular danger zones,” “vascular occlusion,” “biostimulators,” “calcium hydroxylapatite,” “poly‐L‐lactic acid,” “hyaluronan adipogenesis,” and “adipose tissue physiology.”

Articles were selected if they provided primary anatomical data, clinically relevant injectable or surgical anatomy, rheological characterization of HA fillers, safety data relevant to filler injection, biological evidence regarding HA‐tissue interaction, or clinical data on ligament‐line or support‐oriented filler strategies. Priority was given to primary cadaveric anatomical studies, surgical anatomy studies, clinical studies evaluating ligament‐oriented filler strategies, reviews of HA filler rheology, vascular safety publications, and biological studies addressing HA effects on dermal fibroblasts or adipose tissue. Opinion articles were used only when they clarified terminology or current practice patterns. Because the goal of this manuscript was to synthesize a clinically useful framework rather than perform a formal quantitative analysis, no meta‐analysis was attempted. The evidence level of each domain was interpreted qualitatively.

## 3 Repositioning HA Fillers: From Volume Replacement to Support‐Oriented Lifting

The proposed repositioning of HA fillers is not merely semantic. It changes the clinical question from “Where is volume missing?” to “Which anatomical support relationship has failed?” In a volumetric framework, visible depressions, folds, or flattening are often treated by adding filler directly to the area of concern. In a support‐oriented framework, the injector first determines whether the visible change is caused by local volume loss, skeletal deficiency, fat compartment descent, ligament‐associated tethering, dermal laxity, or a combination of these factors. HA is then used only when its immediate mechanical properties can address the dominant anatomical problem.

This shift is particularly important in the midface and lower face, where excessive central filling may worsen facial heaviness, increase facial width, or produce unnatural convexity, whereas selected lateral or deep support may improve contour with less total volume. The purpose of ligament‐associated HA use is not to replace surgery or to claim true ligament regeneration, but to restore a more favorable mechanical relationship between fixed retaining structures, mobile fat compartments, and the overlying soft‐tissue envelope. In this sense, HA fillers should not be abandoned because of overfilled syndrome; rather, they should be redirected from nonselective volume replacement toward selective structural support in anatomically appropriate lifting‐oriented plans.

This framework also reframes the relationship between HA fillers and newer regenerative or biostimulatory procedures. The question is not whether HA fillers are modern or obsolete, but whether they are used for the correct anatomical problem. When the primary concern is diffuse dermal thinning or tissue quality deterioration, a biostimulatory or skin‐quality approach may be more appropriate. When the primary concern is immediate contour‐specific support or support‐related compartment malposition, HA may remain uniquely useful because it can be titrated precisely, reversed when necessary, and used for immediate mechanical effect.

## 4 From Volumization to Structural Support: Why HA Filler Skepticism Requires Reframing

The historical dominance of HA fillers was not accidental. HA fillers provide immediate correction, can be titrated in small increments, are available in a wide range of rheological profiles, and can be enzymatically degraded with hyaluronidase in many clinical situations. These characteristics made HA fillers uniquely suitable for a treatment era in which facial aging was commonly understood as deflation.

Volume loss is undeniably important in facial aging. Skeletal resorption changes the underlying foundation of the face, while deep and superficial fat compartments undergo region‐specific deflation, descent, or redistribution [4, 5, 6, 7, 8]. The identification of discrete facial fat compartments substantially changed the understanding of facial aging and helped explain why generalized or non‐compartmentalized filling may produce unnatural outcomes [4, 5]. When fillers are used thoughtfully to restore appropriate compartmental support or correct focal deflation, HA remains highly useful.

The problem arises when volumization becomes the only framework. Central or medial filling applied repeatedly to tissues that have already descended may increase surface convexity without improving tissue position or facial proportion. In some patients, the cumulative effect may be a face that appears fuller but not younger, wider but not better supported, and smoother but less natural [1]. This pattern should not be interpreted simply as a failure of the filler itself. More often, it reflects indication error, sequencing error, excessive volume, or insufficient anatomical analysis.

A more defensible contemporary role for HA fillers therefore requires a distinction between volume replacement and structural support. HA should not be used reflexively to fill every visible depression. Instead, the injector should first ask whether the visible contour change represents true local volume loss, altered skeletal support, fat compartment displacement, ligament‐associated tethering, skin laxity, or a combination of these factors. Only then can the appropriate modality, plane, product, and sequence be selected.

## 5 Facial Retaining Ligaments: Anatomical Concepts Relevant to Injectables

The facial retaining ligaments are fibrous structures that stabilize superficial soft tissues relative to deeper fixed structures. They are important in surgical facial rejuvenation because they define spaces, tether mobile tissues, and influence the ability of soft‐tissue units to be mobilized and redraped [9, 10, 11, 12]. Although the surgical relevance of retaining ligament release is well established, its injectable relevance is different and must be framed precisely.

Retaining structures are not homogeneous. A clinically useful distinction can be made between true osteocutaneous ligaments and supporting ligamentous or septal structures. True retaining ligaments, such as the zygomatic and mandibular ligaments, arise from periosteum and extend toward the dermis or superficial soft tissues [9, 10, 11, 12]. Supporting ligaments or septal adhesions may arise from deep fascia or represent fibrous condensations between compartments rather than direct bone‐to‐skin structures. Alghoul and Codner emphasized that the retaining ligaments are located in relatively constant anatomical positions and separate facial spaces and compartments, with superficial extensions forming septa that help define subcutaneous fat compartments [12].

The injectable relevance of these structures is closely related to the layered anatomy of the superficial musculoaponeurotic system (SMAS) and its retaining ligament attachments. Surek emphasized that the SMAS should not be regarded only as a surgical facelift structure, but also as a clinically relevant anatomical layer for filler injection planning [13]. In injectable practice, understanding whether a filler is placed superficial to, within, or deep to the SMAS is essential because each plane differs in mobility, vascular relationships, compartmental behavior, and capacity for mechanical support. This perspective is particularly relevant to ligament‐associated HA use, because the intended effect is not simply volume replacement but modulation of the relationship between fixed retaining structures, mobile fat compartments, and overlying soft tissue. Therefore, ligament‐associated filler placement should be interpreted within a layered anatomical model that includes periosteum, deep fascia, retaining ligaments, SMAS, subcutaneous septa, and dermis rather than as an isolated point injection into a ligament.

The line of ligaments is a clinically useful but simplified organizing concept. It has been described as a lateral facial trajectory connecting the temporal ligamentous adhesion, lateral orbital thickening, zygomatic ligament region, and mandibular ligament region [14, 15]. This line separates the face into zones that may behave differently during filler placement. Medial to the line, filler may more readily produce expansion or volumization of mobile tissue. Lateral to the line, filler placed in deep support zones may have a greater tendency to influence support, contour, and apparent lifting. This concept is clinically attractive, but it should not be interpreted as a rigid anatomical boundary or as a guarantee of lifting.

For injectable treatment planning, it is also important to define the temporal ligamentous adhesion and lateral orbital thickening. The temporal ligamentous adhesion refers to a strong fixation zone in the temporal region where superficial and deep fascial layers are adherent. The lateral orbital thickening represents a dense adhesion or thickening near the lateral orbital rim and periorbital soft tissue. These structures are relevant because they participate in the lateral support system of the upper and midface, but they also lie near vascular and periorbital danger zones. Therefore, their inclusion in filler planning must be paired with careful attention to vascular anatomy and conservative injection principles.

The zygomatic ligament is particularly relevant to the midface. It contributes to fixation of the malar soft tissue envelope and is anatomically related to the suborbicularis oculi fat (SOOF), malar fat compartments, and lateral cheek support. The mandibular ligament is relevant to lower‐face contouring, prejowl depression, and the transition between the labiomandibular crease, jowl, and jawline. However, jowl formation should not be attributed simplistically to laxity of the mandibular ligament alone. Cadaveric and anatomical studies suggest that descent or redistribution of cheek fat compartments and changes in surrounding soft‐tissue relationships also contribute importantly to lower‐face aging [16, 17].

The principal facial retaining structures relevant to HA filler planning are summarized in Table 1.

**TABLE 1:** Facial retaining structures relevant to HA filler planning.

Region	Structure	Anatomical category	Injectable relevance	Main caution
Temporal	Temporal ligamentous adhesion	Adhesion/fixation zone	Lateral upper‐face frame; temporal transition; lateral brow support concept	Superficial temporal vessels; variable fascial planes
Lateral orbit	Lateral orbital thickening	Adhesion/fibrous thickening	Lateral orbital and upper midface support concept	Periorbital vascular connections; thin tissue; edema risk
Midface	Zygomatic ligament	True osteocutaneous ligament	Lateral cheek/SOOF support; support‐first midface strategy	Transverse facial, zygomaticofacial, infraorbital/periorbital vessels
Lateral cheek	Masseteric cutaneous ligaments/septa	Supporting ligament/septal structures	Lateral cheek support and compartment boundary	Facial nerve and parotid‐masseteric vascular structures
Lower face	Mandibular ligament	True osteocutaneous ligament	Prejowl and jawline transition support	Facial artery, inferior labial artery, mental artery, submental artery
Chin/labiomandibular region	Mental/medial mandibular ligamentous structures	Classification varies by anatomical definition	Chin–prejowl–labiomandibular transition	Mental vessels; asymmetry; overprojection

Abbreviation: SOOF, suborbicularis oculi fat.

## 6 Aging of the Ligament–Fat Compartment Unit

Aging of the face should be understood as a multilayered process rather than as isolated deterioration of a single structure. The retaining ligaments do not age in isolation. Their apparent clinical relevance changes in relation to skeletal remodeling, fat compartment behavior, dermal thickness, skin elasticity, and regional soft‐tissue mobility.

Skeletal remodeling alters the foundation on which the soft tissues rest. Age‐related changes in the orbital rim, maxilla, pyriform aperture, and mandible may reduce deep structural support and change the vectors of overlying soft‐tissue descent [7, 8]. Fat compartments also undergo region‐specific changes. Some compartments deflate, some descend, and others become more prominent because of relative displacement or boundary effects [4, 5]. These changes can make ligament‐associated tethering more visible. A fold or groove may therefore represent not only volume loss but also the interaction between mobile descended tissue and relatively fixed retaining structures.

The concept of “ligament laxity” should be invoked cautiously. While clinical aging may create the appearance of weakened ligamentous support, direct evidence that HA fillers biologically rejuvenate or tighten aged facial ligaments is lacking. It may be more anatomically accurate to describe aging in terms of altered relationships within the ligament–fat compartment unit. In this model, the retaining ligaments, septa, fat compartments, skin, and skeletal base interact as a mechanical system. HA filler placed in selected deep support zones may improve the contour and positional relationship of this system, but this effect should not be described as ligament regeneration.

Aging status also affects treatment response. Younger patients with early contour changes, preserved skin elasticity, and limited compartment descent may respond to small‐volume, support‐oriented treatment with subtle improvement in contour or facial proportion. Older patients with advanced laxity, severe skeletal resorption, marked soft‐tissue descent, or poor dermal elasticity may require larger structural correction, surgical lifting, or combined treatment. In such patients, attempting to correct advanced ptosis with HA alone may increase facial heaviness and raise the risk of overfilling. Therefore, the same ligament‐associated injection concept cannot be applied uniformly across ages or tissue conditions.

## 7 What Is the Real Target in “Ligament‐Associated” HA Injection?

The term “ligament‐targeted injection” is potentially misleading unless carefully defined. In this review, ligament‐associated HA injection does not mean injection into the ligament substance itself. It also does not imply that HA fillers directly regenerate, shorten, or biologically strengthen the retaining ligaments.

The clinically relevant target is better understood as the ligament‐associated support zone. This may include: (1) the deep periosteal region near the bony origin or insertion of a true osteocutaneous ligament; (2) the periligamentous connective‐tissue condensation adjacent to a retaining ligament; (3) the boundary region between ligamentous septa and adjacent fat compartments; or (4) a deep fat or SOOF‐related support plane adjacent to a retaining structure.

The intended mechanism is mechanical rather than primarily biological. HA filler can create localized projection, improve load‐bearing support, and modify the contour relationship between fixed and mobile soft‐tissue units. In selected regions, this may reduce the need for medial or central filling. However, because injected HA occupies space and changes three‐dimensional surface geometry, any observed lifting effect may reflect a combination of support, projection, contour smoothing, and optical improvement rather than true anatomical repositioning.

This distinction is important because it helps avoid mechanistic overstatement. Deep‐plane facelift surgery releases retaining ligaments to mobilize tissue and permit redraping. HA filler injection does not release the ligament. Therefore, injectable ligament‐associated treatment should not be described as a nonsurgical equivalent of surgical release‐and‐redrape. A more accurate analogy is scaffold augmentation or periligamentous reinforcement. The goal is not to free the soft tissue from tethering, but to improve support at selected structural points.

## 8 Clinical Evidence for Ligament‐Oriented HA Filler Strategies and Its Limitations

Clinical interest in ligament‐oriented filler strategies has been stimulated by the line‐of‐ligaments concept and studies evaluating lateral support‐first approaches. Casabona and colleagues reported that prioritizing injection points lateral to the line of ligaments reduced the filler volume required to achieve aesthetic improvement in the medial midface [14]. In a subsequent study, the authors evaluated a protocol incorporating the line of ligaments and surface‐volume coefficient, reporting measurable changes in midfacial distances after filler placement near the lateral SOOF and zygomatic ligament region [15]. Cong and colleagues combined cadaveric dissection with a clinical technique targeting true facial ligament sites and reported quantifiable lifting effects at corresponding surface landmarks [18].

These studies provide clinically relevant support for the plausibility of support‐oriented filler placement, particularly in the lateral midface. However, the evidence should be interpreted cautiously. Much of the available clinical evidence relies on surface‐distance measurements, three‐dimensional surface imaging, before‐and‐after photographic assessment, or observational technique outcomes. These methods are valuable but cannot definitively distinguish true soft‐tissue repositioning from increased projection, local contour expansion, or changes in surface reflection and geometry. Therefore, statements such as “structural repositioning” should be qualified.

A more evidence‐concordant interpretation is that ligament‐associated HA filler placement may produce support‐mediated contour improvement or apparent lifting in selected patients. The effect is likely region‐dependent, product‐dependent, plane‐dependent, and patient‐dependent. The current literature does not establish a universal algorithm, does not prove long‐term ligament strengthening, and does not demonstrate equivalence to surgical lifting. Future studies should incorporate standardized three‐dimensional imaging, ultrasound or magnetic resonance imaging, validated outcome scales, volume‐controlled comparisons, and long‐term follow‐up to clarify mechanism and durability.

The evidence across these domains is summarized in Table 2.

**TABLE 2:** Evidence map for ligament‐associated HA filler use.

Evidence domain	Representative sources	Strength	Limitation
Retaining ligament and SMAS anatomy	Furnas; Stuzin; Mendelson; Alghoul; Surek; Wong	Strong anatomical and layered‐plane foundation	Surgical and cadaveric anatomy do not directly prove injectable outcome
Fat compartment anatomy	Rohrich and Pessa; Wan et al.	Strong anatomical relevance	Compartment behavior varies with age and individual anatomy
Aging of skeletal/ligament support	Mendelson and Wong; Wong and Mendelson	Strong conceptual relevance	Clinical translation requires individualized assessment
Ligament‐line clinical filler studies	Casabona et al.; Cong et al.	Clinically relevant, technique‐oriented	Mostly observational; surface measurements cannot prove true repositioning
HA rheology	Fundarò et al.; de la Guardia et al.	Useful for product selection	Rheology does not directly equal clinical performance
Vascular safety and safe‐zone anatomy	Wollina and Goldman; Cotofana et al.; Isaac et al.	Essential region‐ and plane‐based safety framework	Vascular anatomy varies; no completely safe zone
HA biology	Wang et al.; Zhu et al.	Supports biological plausibility	Does not prove ligament or facial fat regeneration

## 9 HA Filler Selection: Rheology, Plane, and Volume

Reviewer and practitioner interpretation of ligament‐associated HA injection depends heavily on product selection. It is not appropriate to state that all ligament‐associated sites require the same high G' HA filler. G' or elastic modulus is important because it reflects the ability of a gel to resist deformation under oscillatory shear. In general, fillers with higher elastic modulus may provide greater projection or support in deep structural planes. However, G' alone does not determine clinical behavior [19, 20].

Other properties are also relevant, including viscosity, cohesivity, HA concentration, degree and type of cross‐linking, swelling tendency, extrusion force, tissue integration, and reversibility [19, 20]. A filler with high G' but excessive swelling tendency may be undesirable in thin‐skinned or periorbital regions. A highly cohesive product may remain localized and provide structural support, but excessive firmness or overcorrection may create palpability or unnatural contour. A softer HA filler may be appropriate for superficial refinement but inadequate for deep periosteal support.

Plane selection is equally important. Deep supraperiosteal placement near robust bony support zones may tolerate firmer, more elastic, cohesive HA products, particularly in the lateral cheek, zygomatic, chin, or mandibular support regions. In contrast, the lateral orbital and tear trough‐adjacent regions require conservative product selection because thin tissue, lymphatic sensitivity, edema risk, and vascular complexity reduce the margin of safety. In superficial planes, softer and more tissue‐integrative HA products are generally preferred, and the goal should be refinement rather than structural support.

Volume should be individualized and conservative. The purpose of support‐first treatment is not to place large filler boluses into ligament‐associated regions. Rather, small aliquots placed strategically may reduce the need for larger medial filling. Excessive volume at deep support points can still produce projection, widening, vascular compression, or unnatural contour. In younger patients or early aging, very small volumes may be sufficient. In advanced aging, filler volume should not be escalated simply to compensate for poor surgical indications.

Key rheological and product‐selection considerations are summarized in Table 3.

**TABLE 3:** Rheological and product‐selection considerations for ligament‐associated HA use.

Parameter	Meaning	Clinical relevance	Limitation
G'/elastic modulus	Resistance to deformation under oscillatory shear	Higher G' may support projection and deep structural support	Not sufficient alone to predict lifting; varies by testing method
G”/viscous modulus	Energy dissipation behavior	Influences flow and deformation	Less commonly used clinically
Cohesivity	Ability of gel to remain integrated	May affect localization and structural behavior	No universally standardized clinical threshold
Viscosity	Resistance to flow	Influences injection force and spread	Shear‐dependent; does not equal clinical support
HA concentration	Amount of HA per mL	May affect hydration, swelling, durability	Higher concentration may increase edema risk in sensitive areas
Cross‐linking technology	Gel network architecture	Influences durability, integration, reversibility	Product‐specific and not interchangeable
Swelling tendency	Water uptake after injection	Important in periorbital and thin‐skinned regions	Often underreported clinically
Reversibility	Responsiveness to hyaluronidase	Key safety advantage of HA	Varies by product and clinical setting

## 10 What This Framework Changes in Practice

This framework is intended to change clinical reasoning rather than prescribe a fixed injection recipe. First, treatment planning should not begin with the visible depression alone; it should begin with analysis of the support system that created the visible contour change. A nasolabial fold, tear trough‐adjacent hollow, prejowl depression, or labiomandibular crease may be a local volume problem, but it may also reflect lateral support deficiency, skeletal deficiency, fat compartment descent, ligament‐associated tethering, or dermal laxity.

Second, support should be considered before central filling when the dominant problem is structural. This principle is especially relevant to the midface, where repeated medial cheek or nasolabial filling may increase fullness without improving facial support. In appropriate patients, evaluating and addressing lateral zygomatic or SOOF‐related support before central volumization may reduce total filler volume and produce a more natural contour relationship.

Third, HA should be selected when immediate mechanical support, precise contour modification, or reversibility is clinically important. If the dominant problem is diffuse tissue quality deterioration, skin thinning, or generalized laxity, biostimulatory or skin‐quality modalities may be more appropriate either alone or in combination.

Fourth, product selection should be based on plane and function rather than on the phrase “high G'” alone. Deep support, superficial refinement, periorbital transition, and lower‐face contouring require different rheological behavior and different safety margins.

Fifth, ligament anatomy should not be used to justify more aggressive filler use. A ligament‐based framework is not a rationale for injecting more volume into named anatomical points. It is a rationale for using less filler more intelligently, with clearer anatomical intent, more conservative sequencing, and greater attention to vascular safety.

The proposed decision‐making sequence is summarized in Table 4.

**TABLE 4:** Decision‐making algorithm for repositioning HA fillers from volumization to support‐oriented treatment.

Step	Clinical question	Interpretation	Treatment implication
1	Is the visible defect caused by true volume loss or support failure?	Not all depressions, folds, or shadows are volume deficits	Avoid reflexive local filling; analyze the support system first
2	Is the dominant problem structural, biological, or mixed?	Structural support, tissue quality deterioration, and volume loss require different strategies	Consider HA, biostimulator, skin‐quality treatment, surgery, or staged combination
3	Is lateral or deep support deficient?	Especially relevant in the midface and lower face	Consider support‐first HA strategy before central/medial volumization
4	What is the intended target?	Periosteal/periligamentous support zone, not the ligament substance itself	Use anatomical intent without implying ligament regeneration or direct ligament injection
5	What product behavior is needed?	Projection, support, integration, refinement, reversibility, and swelling behavior differ by product	Select by plane, function, G', cohesivity, viscosity, swelling tendency, and safety margin
6	What are the region‐specific vascular risks?	A structurally logical point is not automatically vascularly safe	Use conservative aliquots, low pressure, slow delivery, reassessment, and hyaluronidase readiness
7	Is the patient an appropriate filler candidate?	Severe laxity, major descent, or poor tissue quality may not respond to HA support alone	Avoid escalating volume; consider surgery, biostimulation, energy‐based treatment, or no treatment
8	How should success be judged?	Aesthetic success is not maximal fullness but improved support, contour, and naturalness	Aim for less reflexive filling, more structural analysis, and better‐directed HA use

## 11 Region‐Specific Decision Framework: Goal and Strategy Rather Than Injection Recipe

The following regional framework is intended as a decision‐making guide rather than a prescriptive injection map. Exact injection points, depth, needle or cannula choice, product selection, and volume must be individualized according to anatomy, prior procedures, vascular risk, tissue quality, and injector expertise. The emphasis is on goal and strategy: what problem is being treated, what support relationship is deficient, and how HA can be used to reduce reflexive filling.

### 11.1 Upper Face and Temporal–Lateral Orbital Region

In the traditional volumetric approach, temporal hollowing or lateral orbital hollowing may be treated primarily by filling the apparent depression. In the support‐oriented framework, the clinical goal is broader: to restore the continuity of the upper lateral facial frame while respecting the temporal ligamentous adhesion, lateral orbital thickening, periorbital tissue thinness, and vascular risk. HA may be considered when immediate contour‐specific support or frame restoration is needed, but the strategy should remain conservative because the temporal and lateral orbital regions contain important vascular structures and communicate with periorbital vascular networks. In this region, the ligament‐associated concept is best understood as lateral frame support rather than direct ligament augmentation.

### 11.2 Midface

In the traditional volumetric approach, midfacial aging has often been treated by directly filling visible medial cheek flattening, nasolabial fold deepening, or tear trough‐adjacent hollowness. Although this approach may improve local fullness, repeated central filling can also increase facial heaviness and width when the dominant problem is loss of lateral support rather than true medial volume deficiency. In the proposed support‐oriented framework, the first clinical question is not how much volume should be added medially, but whether the lateral zygomatic and SOOF‐related support system is deficient. When appropriate, restoring lateral structural support around the zygomatic ligament region may reduce the need for central volumization and create a more favorable lifting‐oriented vector. The goal is therefore not aggressive lateral bolus injection, but strategic support that improves the relationship between fixed lateral retaining structures and mobile medial fat compartments.

### 11.3 Lower Face and Jawline

In the traditional volumetric approach, prejowl depression, marionette shadowing, and jawline irregularity may be treated by filling the visible contour defect directly. In the support‐oriented framework, the clinician first determines whether the defect reflects local volume loss, mandibular skeletal support deficiency, cheek fat descent, chin inadequacy, ligament‐associated tethering, skin laxity, or a combination of these factors. HA may be useful when the goal is to improve transition zones around the chin, prejowl, mandibular border, or jawline. However, the target is not the mandibular ligament substance itself; it is the surrounding support unit and contour transition. Because the lower face is vascularly complex, particularly around the facial, inferior labial, mental, and submental arteries, structural intent must be paired with a conservative technique and complication preparedness.

## 12 Vascular Safety Considerations

A ligament‐associated HA filler review must address vascular safety. Retaining ligaments and ligament‐associated support zones are often located near important vascular structures. Therefore, anatomical precision should not be interpreted as absence of risk. There are no universally safe facial filler zones, and vascular anatomy varies among individuals [21, 22, 23].

Recent vascular anatomy literature has shifted the discussion from generalized danger zones toward region‐specific vascular safe zones and plane‐based risk reduction. Cotofana and colleagues described vascular safe zones for facial soft‐tissue filler injections by analyzing the course, depth, and branching patterns of facial arteries across commonly injected regions [22]. This approach is particularly relevant to ligament‐associated HA filler placement because many proposed support points are located near retaining structures that also serve as anatomical boundaries for vessels, septa, and mobile soft‐tissue compartments. Accordingly, the concept of a ligament‐associated support zone should never be separated from vascular anatomy. A structurally logical injection site is not necessarily a vascularly safe point, and the apparent precision of a ligament‐based approach should not be interpreted as protection from intravascular injection, vascular compression, or embolic complications.

Region‐specific vascular risk should be explicitly considered when applying a ligament‐associated filler strategy. In the zygomatic and lateral midface region, the transverse facial artery, zygomaticofacial vessels, infraorbital branches, and periorbital anastomoses may be relevant depending on injection plane and individual anatomy. In the lateral orbital and SOOF‐related region, vascular communication with the orbital circulation and the thin soft‐tissue envelope substantially narrow the safety margin. In the lower face, injections near the mandibular ligament, prejowl sulcus, chin, and jawline must account for the facial artery as it crosses the mandible, as well as the inferior labial, mental, and submental arterial systems. These anatomical considerations support the use of conservative aliquots, low‐pressure injection, slow delivery, continuous reassessment, and readiness to treat vascular compromise when HA fillers are used.

Risk reduction depends on a combination of anatomy, technique, product selection, and preparedness. General principles include careful patient selection, knowledge of vascular anatomy and its variation, use of small aliquots, low injection pressure, slow injection, avoidance of excessive bolus volume in high‐risk areas, frequent reassessment, and immediate availability of hyaluronidase when HA fillers are used. Aspiration may be performed by some injectors, but it should not be relied upon as a guarantee of safety because false‐negative aspiration can occur. Cannulas may reduce but do not eliminate vascular risk, and needles may provide precision but require careful control of depth and pressure. Ultrasound guidance may be useful in selected high‐risk, revision, or anatomically complex cases, particularly for identifying vessels or previously injected filler.

## 13 HA Fillers and Biostimulators: Complementary but Overlapping Modalities

HA fillers and biostimulators are often framed as competing modalities, with HA representing volume and biostimulators representing biology. This dichotomy is clinically useful only to a limited extent. A more accurate distinction is that HA fillers and biostimulators differ in dominant clinical function, timing of effect, reversibility, and mechanism, while their roles partially overlap.

HA fillers predominantly provide immediate, reversible, contour‐specific structural modification. They are particularly useful when the treatment goal is precise correction of contour, deep support, compartment‐specific volume restoration, or refinement in areas where reversibility is valuable. HA may also induce limited secondary collagen‐related responses, as discussed below; however, this does not make HA equivalent to a classic biostimulator, nor does it establish ligament regeneration.

Biostimulators, including calcium hydroxylapatite (CaHA), poly‐L‐lactic acid (PLLA), and related materials, predominantly provide delayed collagen‐mediated tissue remodeling and improvement in tissue firmness or quality [24, 25]. However, they are not purely biological agents. CaHA, in particular, can be used for facial definition, contouring, and structural support depending on dilution, placement, and indication [26]. Therefore, assigning “architecture” exclusively to HA and “biology” exclusively to biostimulators oversimplifies clinical reality.

The practical question is not which modality is superior, but which anatomical and biological problem is dominant. A patient with focal support deficiency and good skin quality may benefit from HA‐based structural support. A patient with diffuse dermal thinning, laxity, and poor tissue quality may benefit from biostimulation. Many patients have both problems and may require staged or combined strategies. In such cases, HA may provide precise support while biostimulators improve broader tissue quality. Treatment sequence should be individualized, with attention to edema risk, inflammatory potential, reversibility, and future surgical considerations.

## 14 HA Biology, Mechanotransduction, and Adipose Tissue: Supporting but Not Replacing the Mechanical Thesis

HA is not biologically inert. It is a major component of the extracellular matrix and interacts with cell‐surface receptors such as CD44. In photodamaged human skin, cross‐linked HA filler injection has been shown to stimulate de novo collagen production, probably through restoration of mechanical tension and fibroblast activation [27]. These findings support the broader concept that HA filler effects are not limited to passive volume expansion, most plausibly through mechanotransduction rather than a distinct pharmacologic pathway. However, they should not be extrapolated to claim ligament regeneration, fat regeneration, or definitive long‐term lifting. In support‐oriented treatment, the primary clinical effect remains mechanical reinforcement; any collagen‐mediated tissue response should be regarded as a potential secondary contribution to tissue maintenance rather than the principal mechanism of lifting.

HA is also relevant to adipose tissue biology. Hyaluronan participates in adipogenesis, adipose extracellular matrix organization, and adipose tissue remodeling. Experimental literature suggests that HA‐based scaffolds may influence adipose‐derived stem cell differentiation under certain conditions [28]. However, whether injected cross‐linked HA fillers meaningfully modulate facial adipocyte physiology in vivo remains uncertain. Current evidence does not justify claiming that HA filler injection regenerates facial fat compartments or reverses adipose aging in a clinically established manner. Therefore, adipocyte physiology should be discussed as a biological consideration and future research direction, not as a central explanation for lifting‐oriented HA use.

## 15 Preventive Aesthetics: A Hypothesis‐Generating Framework

The increasing interest in prejuvenation reflects a broader shift from correction of advanced aging toward earlier maintenance of structure and tissue quality [29]. In this context, support‐oriented HA filler use may have a potential preventive role, but this concept must be approached carefully.

A preventive framework does not mean placing filler in young patients without clear indication. Rather, it suggests that early anatomical changes may be treated with small, conservative interventions aimed at preserving proportion, avoiding compensatory overfilling, and maintaining facial harmony. In a patient with early lateral midface support loss, subtle HA placement in a structural support zone may reduce the perceived need for repeated medial cheek filling. In a patient with early dermal thinning or reduced firmness, a biostimulator or skin‐quality treatment may be more appropriate than structural HA.

At present, preventive ligament‐associated HA treatment remains hypothesis‐generating. Longitudinal studies are needed to determine whether early support‐oriented treatment changes the trajectory of visible aging, reduces cumulative filler volume, or improves long‐term naturalness. Until such evidence is available, prevention should not become a broad justification for expanding treatment indications. The guiding principle should be anatomical restraint: treat only clearly identified deficits, use the minimum effective intervention, and avoid converting normal aging variation into pathology.

## 16 Evidence Summary and Clinical Implications

The available evidence supports several practical conclusions. First, facial aging should not be reduced to volume loss. Structural support, skeletal foundation, fat compartment behavior, ligament‐associated boundaries, and skin quality must all be assessed. Second, HA filler failure is often a planning failure rather than a material failure. When HA is used to fill medially displaced or unsupported tissues without addressing structural context, the risk of overfilling and unnatural heaviness increases.

Third, ligament‐associated HA injection should be defined precisely. The target is not the ligament substance itself, but a support zone related to periosteal attachments, periligamentous connective tissue, or adjacent fat compartment boundaries. Fourth, the mechanism should be described as mechanical scaffold augmentation, not ligament release, ligament regeneration, or surgical‐like redraping.

Fifth, product selection must be region‐ and plane‐specific. High G' may be useful in selected deep support zones but is not a universal requirement and should not be used as the sole product‐selection criterion. Sixth, vascular safety is central. Ligament‐associated support zones may lie near important vessels, and no injection strategy should be presented without safety considerations.

Finally, HA fillers and biostimulators should be used as complementary, overlapping modalities. HA is often better suited for immediate, precise, reversible structural modification, while biostimulators are often better suited for delayed tissue‐quality improvement. Both domains overlap, and combined strategies may be rational when guided by anatomy and patient‐specific aging patterns.

## 17 Limitations

This review has several limitations. First, it is a narrative review and does not include a formal systematic search, quality scoring, or meta‐analysis. Second, the clinical evidence for ligament‐associated HA filler injection remains limited compared with the broader literature on HA volumization. Third, many available studies use surface imaging, photographic analysis, or observational outcomes, which cannot definitively distinguish true tissue repositioning from projection or contour expansion. Fourth, the rheological behavior of HA fillers varies across products and manufacturing technologies, and published rheological parameters may not translate directly into clinical lifting capacity. Fifth, vascular anatomy varies significantly between individuals; therefore, regional safety principles cannot replace injector expertise, anatomical training, or complication preparedness.

These limitations do not invalidate the anatomical framework, but they require moderation in clinical claims. Ligament‐associated HA use should be presented as an evidence‐informed strategy for selected patients rather than a universally proven lifting method. The aim of this review is therefore not to standardize injection techniques or dosages, but to clarify a treatment paradigm: HA fillers should be used less reflexively as volume replacement agents and more deliberately as anatomy‐guided support tools when structural support is the dominant clinical problem.

## 18 Conclusions

The contemporary skepticism surrounding HA fillers should be interpreted carefully. Many unsatisfactory HA outcomes reflect not an inherent failure of the filler as a product class, but the limitations of applying volumetric thinking to structural and support‐related problems. Facial aging is multilayered, involving skeletal remodeling, fat compartment changes, skin quality deterioration, and altered ligament‐associated support. A more anatomically disciplined framework is therefore needed.

Facial retaining ligament anatomy provides one such framework, but it must be applied precisely. Ligament‐associated HA filler injection should not be understood as injection into the ligament itself, biological ligament regeneration, or nonsurgical replication of surgical ligament release. Rather, it is best understood as mechanical scaffold augmentation in selected deep periosteal or periligamentous support zones. When used conservatively, with appropriate product selection and vascular safety awareness, HA fillers may provide immediate structural support and reduce the need for excessive central volumization in selected patients.

HA fillers and biostimulators should not be viewed as mutually exclusive or hierarchically superior modalities. They differ in dominant function, timing, reversibility, and mechanism, but their roles overlap. The future of injectable facial rejuvenation is therefore not a choice between HA and biostimulators, but a more precise matching of modality to anatomical problem: support where support is deficient, biology where tissue quality is deficient, and restraint where treatment is not clearly indicated.

The decline of indiscriminate volumization should not be mistaken for the decline of HA fillers. Rather, it marks the need to reposition HA fillers within a more anatomically disciplined treatment philosophy. When used as simple volume replacement, HA can contribute to overfilling and facial heaviness. When used selectively as an anatomy‐guided structural support tool, HA remains highly relevant in contemporary facial rejuvenation. The future of HA fillers is therefore not less HA, but better‐directed HA: less reflexive filling, more structural analysis, and more precise support‐oriented treatment planning.

## Author Contributions

Tara Margarella and Seung Jun Shin contributed equally to the conception of the article, interpretation of the literature, drafting of the manuscript, critical revision of the text, and approval of the final version for submission.

## Funding

The authors have nothing to report.

## Ethics Statement

The authors have nothing to report.

## Conflicts of Interest

Both authors hold Medical Affairs positions within the Hugel group of companies: Tara Margarella, M.D. (Hugel Aesthetics, United States) and Seung Jun Shin, M.D., Ph.D. (Hugel Inc., Republic of Korea). The authors acknowledge that Hugel manufactures hyaluronic acid filler and botulinum toxin products; however, no specific Hugel products are discussed, endorsed, or referenced in this article. The arguments presented apply to the HA filler class as a whole and are grounded exclusively in peer‐reviewed literature from independent research groups. The views expressed are the authors' own and do not necessarily represent the official views or policies of their employers.

## Data Availability

No new data were generated or analyzed in support of this article.
